# Exploring the Impact of Saccharin on Neovascular Age-Related Macular Degeneration: A Comprehensive Study in Patients and Mice

**DOI:** 10.1167/iovs.65.4.5

**Published:** 2024-04-01

**Authors:** Steffen E. Künzel, Inga-Marie Pompös, Leonie T. M. Flesch, Dominik P. Frentzel, Vitus A. Knecht, Silvia Winkler, Sergej Skosyrski, Anne Rübsam, Felix Dreher, Norbert Kociok, Moritz Schütte, Alexandre Dubrac, Bodo Lange, Marie-Laure Yaspo, Hans Lehrach, Olaf Strauß, Antonia M. Joussen, Oliver Zeitz

**Affiliations:** 1 Charité–Universitätsmedizin Berlin, corporate member of Freie Universität Berlin and Humboldt–Universität zu Berlin, Department of Ophthalmology, Hindenburgdamm 30, Berlin, Germany; 2 Experimental Ophthalmology, Department of Ophthalmology, Charité–Universitätsmedizin Berlin, Corporate Member of Freie Universität, Berlin Institute of Health, Humboldt-University, Berlin, Germany; 3 Alacris Theranostics, Max-Planck-Straße 3, Berlin, Germany; 4 Département de Pathologie et Biologie Cellulaire, Université de Montréal, Montréal, Quebec, Canada; 5 Max-Planck-Institute for Molecular Genetics, Ihnestrasse 63-73, Berlin, Germany

**Keywords:** neovascular age-related macular degeneration (nAMD), saccharin, artificial sweetener (AS), modifiable risk factors, intravitreal anti-VEGF treatment, treatment need, systems biology, saccharin, choroidal neovascularization (CNV), inflammation, VEGF, complement factors

## Abstract

**Purpose:**

We aimed to determine the impact of artificial sweeteners (AS), especially saccharin, on the progression and treatment efficacy of patients with neovascular age-related macular degeneration (nAMD) under anti-vascular endothelial growth factor (anti-VEGF-A) treatment.

**Methods:**

In a cross-sectional study involving 46 patients with nAMD undergoing intravitreal anti-VEGF therapy, 6 AS metabolites were detected in peripheral blood using liquid chromatography - tandem mass spectrometry (LC-MS/MS). Disease features were statistically tested against these metabolite levels. Additionally, a murine choroidal neovascularization (CNV) model, induced by laser, was used to evaluate the effects of orally administered saccharin, assessing both imaging outcomes and gene expression patterns. Polymerase chain reaction (PCR) methods were used to evaluate functional expression of sweet taste receptors in a retinal pigment epithelium (RPE) cell line.

**Results:**

Saccharin levels in blood were significantly higher in patients with well-controlled CNV activity (*P* = 0.004) and those without subretinal hyper-reflective material (*P* = 0.015). In the murine model, saccharin-treated mice exhibited fewer leaking laser scars, lesser occurrence of bleeding, smaller fibrotic areas (*P* < 0.05), and a 40% decrease in mononuclear phagocyte accumulation (*P* = 0.06). Gene analysis indicated downregulation of inflammatory and VEGFR-1 response genes in the treated animals. Human RPE cells expressed taste receptor type 1 member 3 (*TAS1R3*) mRNA and reacted to saccharin stimulation with changes in mRNA expression.

**Conclusions:**

Saccharin appears to play a protective role in patients with nAMD undergoing intravitreal anti-VEGF treatment, aiding in better pathological lesion control and scar reduction. The murine study supports this observation, proposing saccharin's potential in mitigating pathological VEGFR-1-induced immune responses potentially via the RPE sensing saccharin in the blood stream.

Age-related macular degeneration (AMD) remains the leading cause of severe vision loss in older people. Intravitreal treatment with anti-vascular endothelial growth factor (anti-VEGF IVT) has revolutionized the outcome of the late, neovascular stage of the disease (nAMD). Despite the undisputed success of this approach, the disease is not yet fully understood, treatment outcomes are far from optimal, and there continues to be a great demand for novel therapeutic and preventive measures.^1^^–^^3^

Although age and genetic variants are by far the strongest but unmodifiable risk factors for late AMD, there is reliable experimental and clinical evidence for environmental factors as disease driving or mitigating conditions.^1^^,^^3^ Several studies have recognized the beneficial role of diet regarding AMD pathogenesis in this context, with certain antioxidants, fish oils, polyunsaturated fatty acids, and decreased vitamin D intake in the scientific and clinical attention.^1^^,^^3^ However, dietary habits are subject to individual, spatial, and ongoing dynamic changes – with more modern but less researched food products potentially impacting clinical disease manifestation. In a worldwide trend toward health and fitness, artificial sweeteners (ASs) emerged as common food additives and sugar substitutes during the last decades.^4^^,^^5^ Thereby, ASs mimic the sweet taste of sugar by factors of up to several hundred with effectively no nutritional value.^6^ Being considered as harmless by competent authorities, ASs are used to sweeten a wide spectrum of food products and for masking the bitter taste of medicines, thereby being of particular importance in aged patient cohorts at risk of comorbidity and polypharmacy; for example, in AMD.^4^^,^^5^^,^^7^^,^^8^ Numerous studies propose associations between AS intake and diseases of multiple organ systems – with AS potentially as both protective and detrimental agents.^9^^–^^11^ In terms of AMD research, knowledge is limited. Promisingly, ASs act as mitigators of VEGF-induced permeability and leakage in the kidneys and the lungs,^12^^,^^13^ and one in vitro study has even reported a potential link between AS and neovascular diseases of the retina with AS attenuating VEGF-induced vasculogenesis.^14^

With this work, we test whether AS blood levels feature on relevant nAMD disease outcomes in terms of morphology and function. Thereby, we match peripheral blood levels of 6 common ASs with clinical nAMD features in a cohort of 46 patients with nAMD under anti-VEGF IVT stratified by the activity of choroidal neovascularization (CNV) into a stratum of chronically active CNV (CAC) and effectively controlled CNV (ECC).

Interestingly, although we observe only a slight impact of cumulative AS blood levels on nAMD features, we identify saccharin as particularly protective, that is, less subjects with saccharin use showed the CAC phenotype of CNV activity. In a translational murine experiment of laser-induced CNV, saccharin acts as a mitigator of pathological VEGFR-1-induced immune responses. In line with the human findings, orally treated mice yield beneficial ocular phenotypes in terms of less leakage and less fibrosis.

## Materials and Methods

### Study Design (BIOMAC)

This study was part of a cross-sectional observational study on nAMD biomarkers at Charité University Hospital, Berlin, Germany. The research protocol was conducted in accordance with the valid versions of the study protocol, ICH Good Clinical Practice Guidelines (ICH-GCP), the tenets of the Declaration of Helsinki and was approved by the competent ethics committee of the Charité University Hospital, Berlin. All included participants provided written informed consent and were recruited prospectively.

### Study Protocol and Subject Recruitment

From November 2018 through June 2020, eligible participants meeting all inclusion and none of the exclusion criteria were enrolled at the time of their regular appointments at the Department of Ophthalmology at Campus Benjamin Franklin (CBF) of Charité University Hospital, Berlin. Charts including imaging results of patients that recently (within preceding 6 month of this study) received anti-VEGF IVT were reviewed retrospectively. Inclusion criteria included both genders ≥51 years of age, active subfoveal CNV secondary to nAMD (all lesion types) in the study eye, BCVA_LogMAR_ ≥ +0.1 and ≤ +1.3 in the study eye (in case both eyes of an individual patient met the inclusion criteria, the eye with the lower visual acuity was included, in case of both eyes having equal visual acuity [VA], the eye with the clearest lens and ocular media and least amount of subfoveal scar or geographic atrophy was selected), and informed written consent. Exclusion criteria included any causes of CNV other than neovascular AMD in the study eye, subretinal hemorrhage in the study eye, which warrants surgical intervention except for intravitreal therapy with anti-VEGF IVT, any contraindication for continuous intravitreal therapy, and any kind of dependency on the investigator or employment by the sponsor or investigator.

As a stratification strategy during the recruiting process, we assigned patients to two distinct cohorts based on their CNV activity under anti-VEGF IVT: CAC versus ECC. Criteria for assignment to the CAC cohort included: IVT intervals between the current and the last as well as between the last and second last intravitreal injection in the study eye was ≤42 days (6 weeks), and CNV was regarded as active in the study eye as evidenced by residual fluid present on optical coherence tomography (OCT) at the current and the last 2 visits before the injections. Assignment criteria for the ECC cohort were: the intervals between the current and the last as well as between the last and second last intravitreal injection in the study eye was ≥70 days (10 weeks), and CNV activity was regarded as controlled in the study eye as evidenced by absent or stable fluid on OCT at the current and the last 2 visits before injections.

### Clinical Examination and Meta-Feature Logging

Visual function of the study eye and the fellow eye were assessed using the Early Treatment Diabetic Retinopathy Study Group 1985 (ETDRS) protocol. All participants received complete bilateral ophthalmologic examination, including a dilated fundus examination. Recruited subjects were bilaterally imaged by fundus autofluorescence imaging (FAF), OCT, fluorescein angiography (FA; all: Spectralis, Heidelberg Engineering, Heidelberg, Germany), and optical coherence tomography angiography (OCT-Angiography; ZEISS Angioplex). Imaging was done by highly experienced technicians following standard procedures to ensure consistency and high quality in image acquisition. For meta-feature annotation in terms of epidemiological (age and sex) and general health features (smoking status, pulmonary dysfunction, history of smoking, diabetes mellitus, arterial hypertension, profession, and medication plans), information was extracted from the electronic patient record. All data relevant to the study were documented soon after measurement by the investigatory team in the clinical software database. Match of meta and proteomic features on individual patient level occurred at later analysis steps (compare below).

### Sample Collection, Preparation, and Mass Spectrometry Analysis

Due to the length of the paragraph describing this methodological approach in detail, we have summarized it in a Supplementary Material 1 file.

### Bioinformatics: Data Extraction, Compound Identification, Curation, Metabolite Quantification, and Block Correction

Due to the length of the paragraph describing this methodological approach in detail, we have summarized it in a Supplementary Material 1 file.

### Murine Experiments

The animal experiments complied with the guidelines of the ARVO statement for the use of animals in ophthalmic and vision research. They were also approved by the local government authorities (Landesamt für Gesundheit und Soziales, LaGeSo, Berlin). Twelve male C57BL/6J mice purchased from Charles River (Germany) have been used for the described experiments. All of them were 10 to 14 weeks old and weighed more than 22 g (mean 25.5 g) at the time of the laser-induced CNV. The animals were housed in a 12-hour day/night cycle and got food and water ad libitum. One day before the laser treatment, we added 0.03% saccharin (Thermo Fisher Scientific, Waltham, MA, USA) to the drinking water of our saccharin intervention group (Sa; *n* = 6). The Sa group got the saccharin-containing water for 15 days (until the end of the experiments) while the control group (Co; *n* = 6) got pure water.

### Laser-Induced CNV and Fluorescence Angiography

For in vivo experiments, we dilated the pupils by using phenylephrin tropicamide eye drops (Charité Apotheke, Berlin, Germany). Subsequently, the mice were anesthetized with a subcutaneous injection of ketamine (100 mg/kg) and xylazine (12 mg/kg). In deeply anesthetized animals, we induced the CNV with an argon ion laser (Visulas 532 s, Carl Zeiss Meditec, Oberkochen, Germany) adjusted to 120 mW, 100 ms, and 50 µm. We placed four spots around the optic nerve head of each eye to perforate the Bruch's membrane without infringing the large vessels. The incidence of bleeding for each laser spot was recorded using a severity grading system ranging from 0 to 2. Additionally, we noted whether the bleeding was due to technical issues for exclusion purposes or was a biological effect, as per the criteria outlined by Gong et al. 2015.^15^ 14 days later, we evaluated the CNV at these spots with FA in anesthetized mice. To detect the new pathological leaky choroidal vessels, we injected fluorescein (5 mg/kg, fluorescein 10%; Alcon, Freiburg, Germany). Five minutes after the subcutaneous injection, we made a fundus angiography (FAG; 488 nm) on a Spectralis HRA-OCT with a 55 degree lens (Heidelberg Engineering, Heidelberg, Germany). After the end of the in vivo experiments, the eyes were treated with Corneregel (Bausch & Lomb GmbH, Berlin, Germany). The leakage area and integrated density (IntDen) we quantified with ImageJ (1.53o, National Institutes of Health, Bethesda, MD, USA).

### Immunohistochemistry Staining of Choroidal Flat-Mounts

After the enucleation, we fixed the eyes for 13 minutes in 4% PFA. By a circular cut, the cornea could be dissected from the fixed eyes to remove the lens and vitreous. With four to five cuts from the peripheral fundus toward the optic nerve, the eyecups were flatted without splitting the CNV spots. After cutting the optic nerve, the retinae could be removed. The remaining part of the flat-mount (containing the retinal pigment epithelium [RPE], choroid, and sclera) was permeabilized by an overnight incubation in 5% Triton X-100 in TBS at 4°C. Subsequently, we blocked the samples for 4 hours with a 5% BSA solution. Then, the RPE cells were stained with phalloidin, together with the common fibrosis marker vimentin and the microglia marker Iba1 (the primary and secondary antibodies are listed in Supplementary Table S2).^43^^,^^44^

The samples were incubated overnight at 4°C with the primary antibodies before we washed them 3 times with TBS to incubate them afterward for 90 minutes with the secondary antibodies at room temperature. At the end, we washed the flat-mounts three times with TBS and mounted them on glass slides with Mounting Fluorescence Medium (DAKO). The flat-mount samples were stored in the dark at 4°C until the examination at the confocal microscope Leica SPE (Leica Microsystems GmbH, Wetzlar, Germany). We used Leica Application Suite X (3.7.4.23463; Leica Microsystems CMS GmbH) to count the Iba1 positive cells (in the area of positive vimentin staining and with irregular cell borders in the RPE layer) and ImageJ to quantify vimentin. The volume of fibrosis was calculated by multiplying the largest area perimeter by the widest thickness of the vimentin-positive area.

### RNA Isolation and Quantitative PCR

After enucleation of the eyecups, the retinae were separated from the choroidal part. The separated samples were snap-frozen in liquid nitrogen before we stored them at −80°C until the isolation. The RNA was extracted using the RNAeasy Plus Mini Kit (Qiagen, Hilden, Germany). The cDNA was obtained using the QuantiTect Reverse Transcription Kit (Qiagen, Hilden, Germany), and gene expression was evaluated by quantitative PCR (qPCR) using the QuantiNova SYBR green PCR kit (Qiagen, Hilden, Germany) on a RotorGene (Qiagen, Hilden, Germany) and analyzed using the ddCT method.

### mRNA Analysis in RPE Cell Line

ARPE-19 cells (ATCC, CRL-2302) were cultured in DMEM/F12 (Cat. No: 11320033; Thermo Fisher, Darmstadt, Germany) supplemented with 10% FBS (Cat. No: 35-015-CF, Corning, Corning, NY, USA) and 1% Streptomycin (Cat. No: BS.A 2213, Bio&SELL, Feucht / Nürnberg, Germany) at 37°C and 5% CO_2_.

The day before the experiment, semi confluent cells were cultured in serum-free medium. The cells were incubated with 0.03% saccharin (Cat. No: 223370010; Thermo Fisher, Darmstadt, Germany) in serum-free medium for 24 hours. RNA-isolation was performed using the RNeasy Plus Mini Kit (Cat. No: 74136; Qiagen, Hilden, Germany), followed by reverse transcription using the QuantiTect Reverse Transcription Kit (Cat. No: 205311; Qiagen, Hilden, Germany). Primers for qPCR were obtained from Eurofins Genomics (Ebersberg, Germany; see Supplementary Table S2). The qPCR was conducted with the Biozym SYBR green PCR kit (Cat. No: 331416S; Biozym, Hess, Oldendorf, Germany) on a RotorGene (Qiagen, Hilden, Germany) and analyzed using the ddCT method. The detection of TAS1R3 expression in ARPE-19 cells was performed by PCR followed by 2% agarose gel electrophoresis with ethidium bromide.

### Data Analysis

#### Animal Model

**Figure 1.**
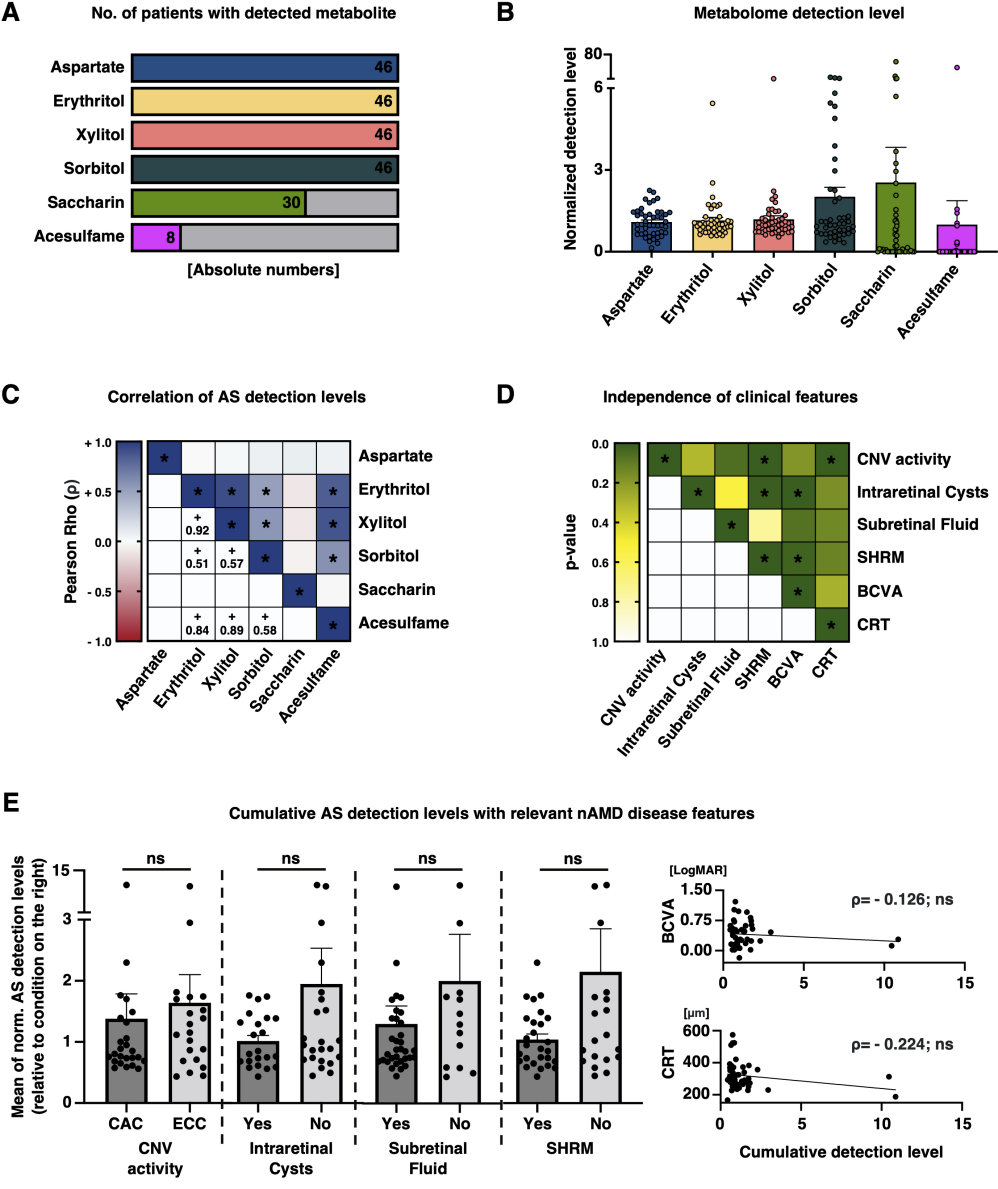
Detection levels of artificial sweetener metabolites in peripheral blood samples and match of mean AS levels with relevant nAMD disease features. (**A**) Visualization of the absolute number of patients with non-zero detection level for indicated AS metabolite. (**B**) Quantitative detection level for indicated AS. Each dot represents one patient. Bars represent means with error bars indicating standard error of the mean (SEM). No statistical testing. Values are normalized to the median of every metabolite and batch following our normalization strategy to prevent batch effects (refer to Supplementary Material 1). (**C**) Correlation analysis for quantitative levels between indicated AS. *Color* indicates Pearson Rho (ρ). *Asterisk* indicates significant correlations (*P* < 0.05). For significant correlations, ρ is indicated in the schematic. (**D**) Independence analysis between relevant meta-features. Fisher's exact test is applied for binary variables. Mann-Whitney *U* test is applied for testing between binary and numeric variables. Pearson's correlation coefficient is used for testing of correlations between two numeric variables. The *P* value is color-coded. The *asterisk* indicates statistical significance. Significant dependencies and differences are precisely described in the manuscript. (**E**) *Left* four diagrams: Scatter plot indicating quantitative detection levels of mean of six AS levels. All *dots* are normalized to mean of condition on the right. The *bars* represent means with error bars indicating SEM. Mann-Whitney *U* test is applied. *Right*: Correlation analysis between mean AS levels and BCVA_LogMAR_ and CRT. Pearson Rho (ρ) is indicated. Both correlations are not significant. *P* < 0.05 is considered statistically significant for all tests in this figure.

**Figure 2.**
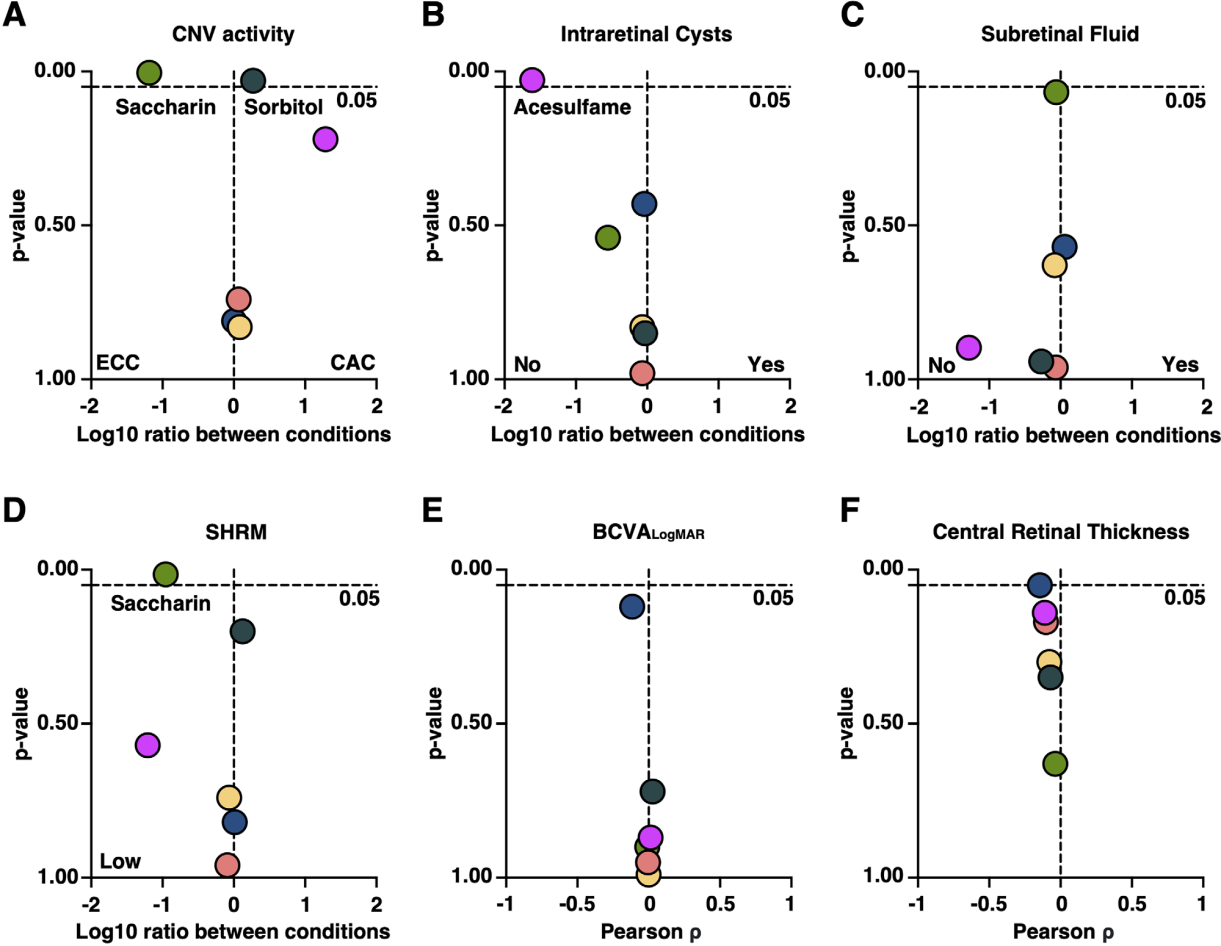
Single AS metabolites are significantly correlated with multiple functional and morphological nAMD disease features. (**A**–**D**) Volcano plot with x-axis: log10 AS detection level ratio of individual AS between indicated conditions, and y-axis: *P* value. Mann-Whitney *U* test is applied. (**E,** **F**) Volcano plot with correlation analysis between individual AS detection levels and numeric features BCVA_LogMAR_ and CRT. X-axis: Pearson ρ, and y-axis: *P* value. Any *P* < 0.05 is considered statistically significant for all tests in this figure. Color-coding refers to individual metabolites: *light-green* = saccharin, *dark-green* = sorbitol, *fuchsia* = acesulfame, *dark-blue* = aspartate, *yellow* = erythritol, and *orange* = xylitol.

**Figure 3.**
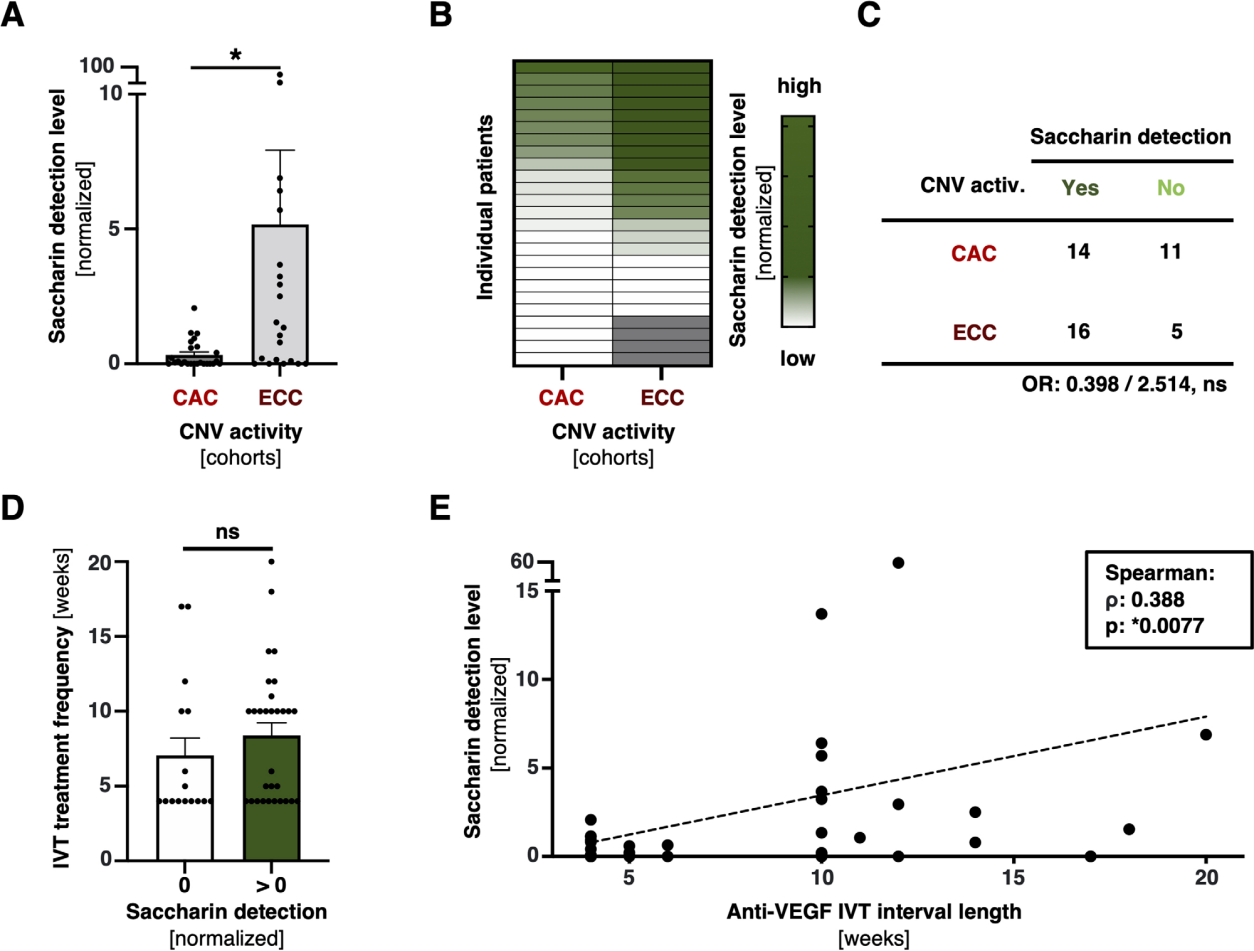
Positive correlation of saccharin blood levels and CNV suppression under anti-VEGF IVT. (**A**) Saccharin detection levels between CAC and ECC patient divisions. Bars represent means with error bars indicating SEM. Mann-Whitney *U* test is applied, with *P* < 0.05 considered statistically significant. (**B**) Heatmap indicating saccharin detection levels between indicated stratification divisions. (**C**) Contingency table between qualitative saccharin detection (yes versus no) and CNV activity cohorts (CAC versus ECC). (**D**) anti-VEGF treatment frequency in weeks between patients with and without saccharin detection. Graph and statistical testing similar to (**A**). (**E**) Correlation analysis between anti-VEGF IVT frequency in weeks and saccharin detection levels. Spearman Rho (ρ) and *P* value is indicated.

Normal distribution of data was tested either by normal distribution confirmed by Shapiro-Wilk + Kolmogorov-Smirnov test or D'Agostino and Pearson test. Groups were compared by unpaired *t*-test (significance given at *P* < 0.05). In the case the test for normal distribution failed for one of the data groups, we did not compare the data by statistic testing but rated by biological significance (e.g. agonist receptor relation). Statistical testing strategy is indicated in the figure legends for the patients with nAMD data (Figs. 1–3).

## Results

### Human Study Population

We recruited 46 patients with nAMD, stratified by CNV activity under anti-VEGF IVT with 54% of participants (*n* = 25) in the chronically active CNV (CAC) compared to 46% in the effectively controlled CNV group (ECC, *n* = 21, refer to the Methods section for details on the stratification strategy and criteria).^16^ As recently reported by us, this study cohort is well-representing the general nAMD population concerning multiple clinical features, including age, gender, and best-corrected visual acuity (BCVA_LogMAR_, Supplementary Table S1).^3^^,^^16^ Expectedly, the two groups differed in functional and morphological parameters reflecting CNV activity, for example, in frequency of anti-VEGF IVT (CAC = 4.32 weeks, w. ± 0.61 w. vs. 12.24 w. ± 3.1 w., mean ± standard deviation, stratification criterion, *P* < 0.0001), central retinal thickness (CRT; CAC = 328.9 µm ± 74.3 µm vs. ECC = 274.8 µm ± 45.7 µm, *P* = 0.0025), and subretinal hyper-reflective material (SHRM; CAC = presence in 21/25 = 84% vs. ECC = 5/21 = 23.8%, *P* < 0.0001). BCVA_LogMAR_ (CAC = 0.48 ± 0.34 vs. ECC = 0.40 ± 0.24, *P* = 0.27) showed a tendency, albeit not being statistically significant, as well as the presence of subretinal fluid (SRF; CAC = 17/25 = 68% vs. ECC = 12/21 = 57.1%, *P* = 0.056), and intraretinal cysts (IRCs; CAC = 13/25 = 52% vs. ECC = 9/21 = 42.9%, *P* = 0.29; see Fig. 1D, Supplementary Table S1). Expectedly, some of these features are not independent for the given patient cohort (see Fig. 1D). SHRM and IRC (18 patients positive for both, 14 negative for both, and only 5 patients with IRC but without SHRM, and 9 with SHRM but no IRC, *P* = 0.004) were not independent. Similarly, patients with IRC had a worse visual outcome than those without (BCVA_LogMAR_: IRC = 0.57 ± 0.25 vs. No-IRC = 0.32 ± 0.26 mean ± standard deviation, *P* = 0.0013) – the same applies to patients with SHRM (with SHRM = 0.51 ± 0.3 vs. 0.35 ± 0.23, *P* = 0.0329).

### Detection of Six Artificial Sweeteners in Peripheral Blood Samples of all Patients

Blood samples of every patient were collected at a single timepoint and subjected to liquid chromatography - tandem mass spectrometry (LC-MS/MS) based metabolomics. We considered six molecules for further analysis including erythritol, xylitol, sorbitol, saccharin, and acesulfame as the AS, and with aspartate as important metabolite of aspartame (see Fig. 1A). Interestingly, aspartate, erythritol, xylitol, and sorbitol were detected in peripheral blood samples of all patients with relatively balanced detection levels for aspartate, erythritol, and xylitol. In contrast, saccharin (30 of 46 samples) and acesulfame (8) were detected in a smaller subset of patients with wider variability in detection levels (see Figs. 1A, 1B). Furthermore, we tested for correlations among the six AS blood levels. Although aspartate and saccharin detection were not significantly correlated with any other AS blood level, erythritol, xylitol, sorbitol, and acesulfame were significantly and positively correlated (see Fig. 1C).

### Cumulative Blood Levels of all Six AS do not Significantly Impact nAMD Disease Features – But Single AS Metabolites do

Subsequently, we investigated significant differences in total AS blood levels (mean of the normalized detection levels of all 6 AS metabolites) among patients with different nAMD manifestations. Interestingly, higher AS levels were detected in patients with more favorable phenotypes characterized by lower chronic disease activity for six clinical nAMD features: CNV activity suppression (CAC vs. ECC), absence of IRCs, absence of SRF, absence of SHRM, reduced BCVA_LogMAR_, and decreased CRT. However, these findings did not reach statistical significance based on the available data (see Fig. 1E).

Next, we investigated significant changes of specific AS metabolites among the mentioned conditions. Significantly higher levels of saccharin (*P* = 0.004), whereas slightly lower levels of sorbitol (*P* = 0.03) were detected in patients with effectively controlled CNV (see Fig. 2A). In addition, higher levels of acesulfame were found in patients without IRC (*P* = 0.028), and higher levels of saccharin were detected in patients without SHRM (*P* = 0.015; refer to Figs. 2B, 2D). No significant dependencies were observed for other nAMD features (refer to Figs. 2C, 2E, 2F).

### Quantitative, But not Qualitative Importance of the Artificial Sweetener Saccharin for Chronic CNV Activity

Saccharin detection levels were higher in patients categorized as ECC (*P* = 0.004; refer to Figs. 2A, 3A, 3B). Among the 16 patients without saccharin detection, 11 fell into the CAC division (refer to Fig. 3C). This yields an odds ratio of 0.398 for patients in this group when saccharin is detected, although it is not statistically significant (*P* = 0.217). In a binary context analysis (saccharin detection: >0 vs. 0), although there is a trend for more frequent anti-VEGF IVT in patients without saccharin detection in their blood, this finding is also not statistically significant (*P* = 0.215; see Fig. 3D). In contrast, robust statistical significance was obtained when performing quantitative correlation analysis between saccharin values and anti-VEGF IVT interval length (see Fig. 3E for the results; *P* = 0.0077, Spearman's rho = 0.3879). These results suggest a quantitative or dose-dependent role of saccharin in this condition, where patients with lower anti-VEGF IVT frequencies exhibited higher levels of saccharin in their blood.

### Beneficial Morphological Phenotype in Saccharin-Fed Animals in a Murine CNV Model

To investigate the potential impact of saccharin on choroidal neovascularization, we conducted an interventional preclinical study using an established mouse model.^17^^,^^18^ The mouse model involves laser-induced rupture of the RPE and choroid, which triggers endothelial proliferation, migration, and a robust inflammatory response. Briefly, mice were subjected to four laser burns around the optic nerve. The analysis was conducted 14 days after laser treatment. The choice of this specific time point was informed by clinical data, as saccharin showed a correlation with extended injection intervals. Therefore, we anticipated a more significant saccharin effect at a later time frame after the laser treatment. Consequently, we opted for the 14-day mark, which represents the longest duration after laser treatment mentioned in standard literature. After 14 days, the laser-treated areas were examined using fluorescence angiography and fundus imaging. Bleeding events were not imaged but meticulously documented and graded on a scale from 0 to 2 for each laser spot, occurrence and severity were finally given in percentage to all laser spots for the 2 groups. Subsequently, the mice were euthanized for immunohistochemical analysis and qPCR. Throughout the experimental period, the control group received water, whereas the treatment group received 0.03% saccharin through their drinking water from the day preceding the laser procedure until day 14.

**Figure 4.**
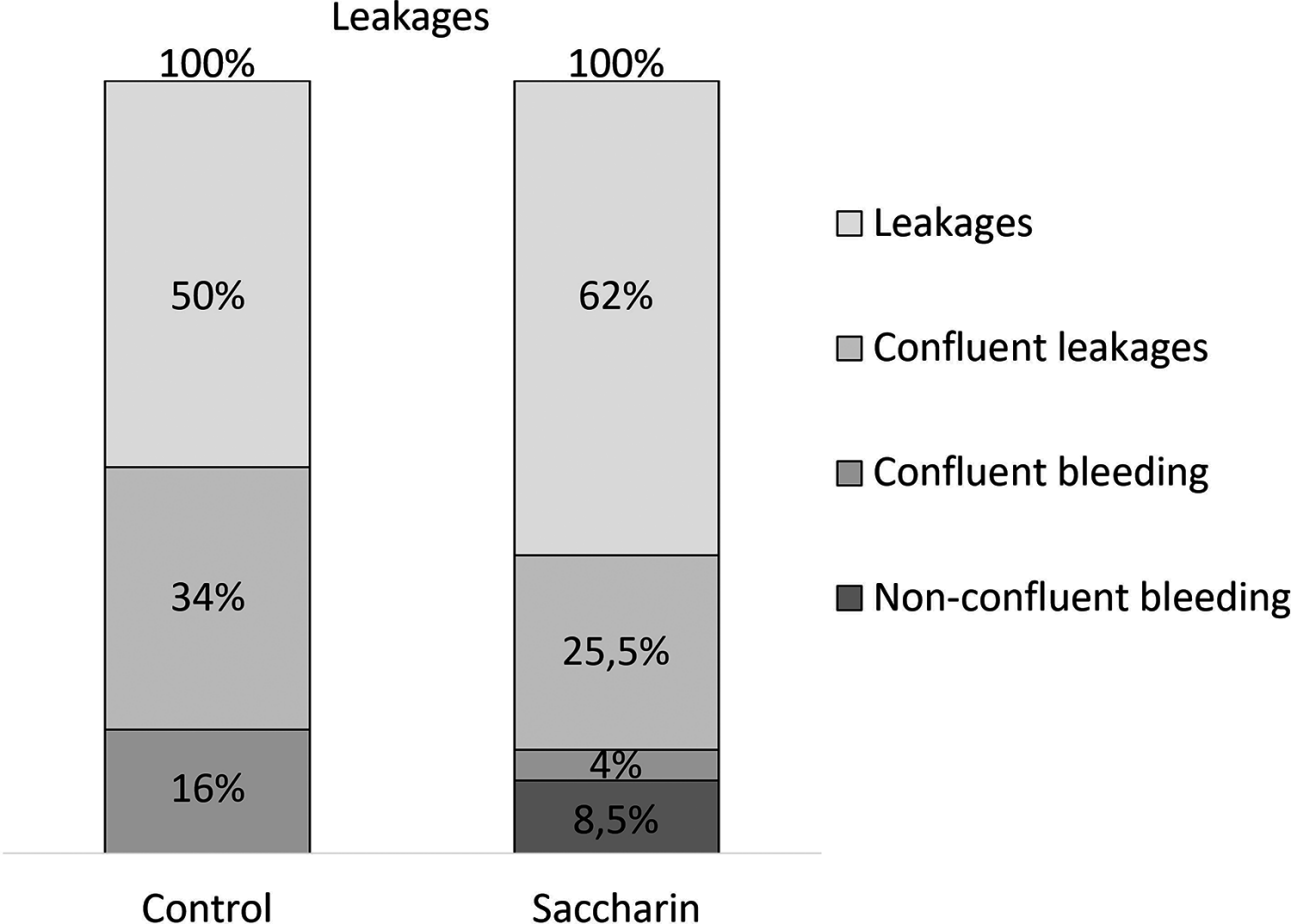
Intervention in the murine CNV model: percentage distribution of CNV lesion phenotypes between the two interventional groups. CNV laser lesions were analyzed by means of fluorescein angiography, 14 days after laser; the fluorescein leakage patterns were categorized into the phenotypic groups: leakage, confluent leakage (fluorescein extravasations of 2 neighboring scars started to mix), additional bleeding and confluent bleeding, graded as 1 or 2 (control *n* = 44 = 100% and saccharin *n* = 47 = 100% with *n*: number of leakage areas).

**Figure 5.**
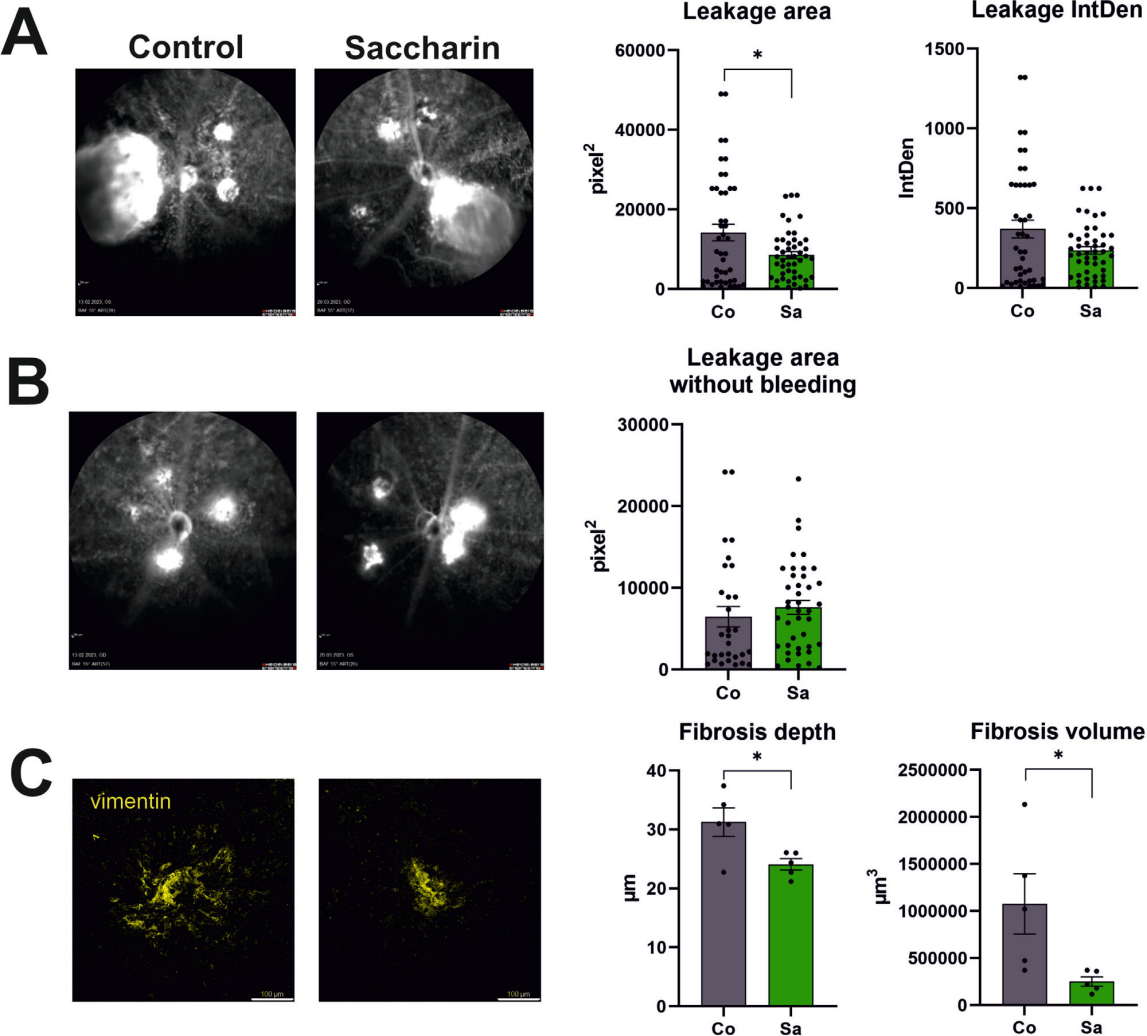
Structural analysis of the lesion areas in laser induced CNV. (**A**) Quantification of fluorescein leakage. *Left panels*: fluorescence fundus photos of laser lesions in control mice and in saccharin-treated mice after 14 days; *right panels*: plotting the leakage area defined as the fluorescent area for comparison between control and saccharin (*P = 0.012; normal distribution tested by the D’Agostino and Pearson test; unpaired *t*-test)**;** integral of fluorescence density (leakage IntDen, in all groups control *n* = 44 and saccharin *n* = 47). (**B**) Quantification of fluorescein leakage in areas without bleeding (control *n* = 30 and saccharin *n* = 41; *n* = number of leakage areas) that appear with sharper delimited borders compared to those from laser spots with preceding bleeding (panel **A**). (**C**) Histologic analysis of scar areas inside the laser CNV lesions: areas are defined by area of vimentin-positive staining: *left panels:* vimentin-positive areas in a flat-mount preparation of an eye in a control mouse and a saccharin-treated mouse; *right panels:* comparison of fibrosis depth and fibrosis volume (measured by z-stacks in confocal microscopy) between control eyes (*n* = 5 fibrotic areas) and eyes of the saccharin group (*n* = 5 fibrotic areas); normal distribution confirmed by the Shapiro-Wilk + Kolmogorov-Smirnov test; significance by unpaired *t*-test: *P = 0.026 (depth) and *P* = 0.03 (volume). Bars represent means with error bars indicating SEM for all graphs in this figure.

In vivo analysis demonstrated notable differences between the two groups (refer to Figs. 4, 5). Although both groups exhibited fluorescein leakage from newly formed blood vessels, this phenotype was less severe in the saccharin group. The saccharin group displayed milder leakages with fewer confluence formations between adjacent laser burns, which also resulted in less convergence of previously bleeding spots (refer to Fig. 4). Quantitative analysis revealed smaller leakage areas and lower amounts of leaked fluorescein, as measured by density integration, in the saccharin group (refer to Figs. 5A, 5B).

Based on these findings, we formulated the hypothesis that the saccharin group exhibited a reduced propensity for wound formation and a faster wound healing process. To test this hypothesis, we examined scar formation at the sites of laser impact using immunohistochemistry with an antibody against vimentin (refer to Fig. 5C). Our analysis revealed that the depth and volume of fibrosis were significantly smaller in the saccharin group compared to the control group.

### Saccharin Attenuates Expression Levels of Inflammatory and VEGF Response Genes Including Complement Factor 3 and 5 in the RPE and Choroid

**Figure 6.**
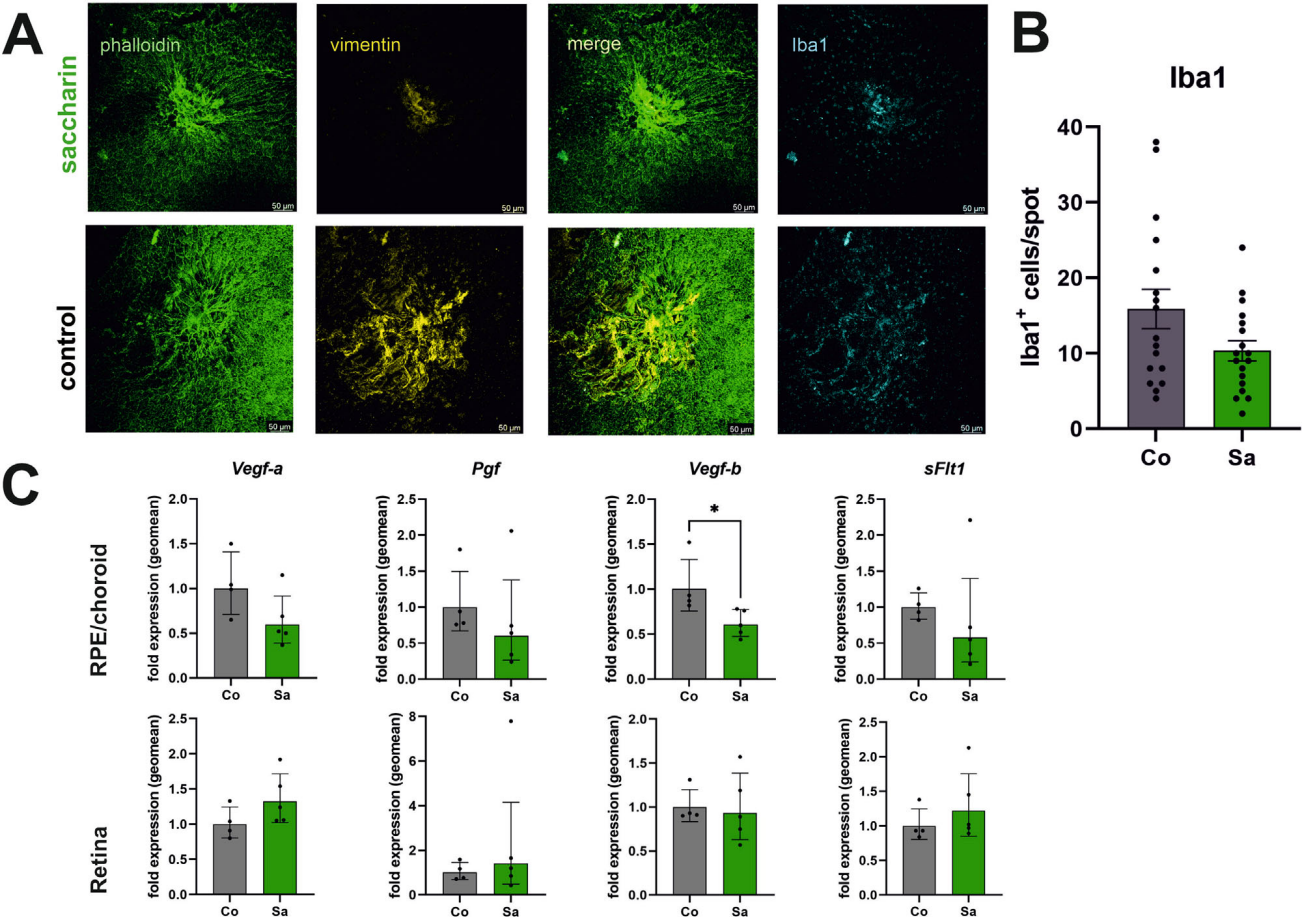
Mechanistic insight into saccharin effects in laser-CNV. (**A**) Immunostaining of laser-CNV areas in choroidal flat-mount preparations of eyes in the saccharin group (*upper row*) and of the control group (*lower row*): anti-phalloidin (*green* = shows actin filaments and highlights the cell border in epithelial cells), anti-vimentin (*yellow* = fibroblasts and fibrosis), and anti-Iba1 (*blue* for mononuclear phagocytes). (**B**) Counts of mononuclear phagocytes (Iba1 positive cells) in the CNV area (defined by vimentin staining and area of irregular RPE cell morphology) for the group control and saccharin (*n* = 17 control and *n* = 18 saccharin with *n* = number of CNV areas); test for normal distribution by the D’Agostino and Pearson test revealed positive; unpaired *t*-test *P* = 0.06. Bars represent means with error bars indicating SEM (**C**) qPCR to determine differences in the gene expression; mRNA was harvested from either RPE/choroid tissue preparations (*upper row*) or retina preparations (*lower row*) of mice from the control group (*n* = 4 retinal or RPE/choroid tissue samples; *grey bars*) and the saccharin group (*n* = 5 retinal or RPE/choroid tissue samples; *green bars*); the figure summarizes the data with biological relevance: only differences in VEGR-1 signaling (*Vegfa*, *Pgf*, and *Vegfb*: *P* = 0.0159, sFlt1) in the RPE/choroid group. Other genes are shown in the supplement. Bars represent geomeans with error bars indicating SD.

In a previous study, we demonstrated that a reduction in neovascularization and scar formation coincided with a decrease in the number of mononuclear phagocytes recruited after laser impact.^19^ Consequently, we formulated a hypothesis suggesting that the saccharin effect could be attributed to a reduction in mononuclear phagocyte migration. To investigate this hypothesis, we examined the presence and quantity of mononuclear phagocytes (Iba1-positive cells) in the scar area defined by vimentin-positive staining (refer to Fig. 6).^20^ Remarkably, we observed a 40% reduction in the number of Iba1-positive cells (*P* = 0.06; refer to Fig. 6A). These findings suggest that alterations in pathways associated with inflammation, fibrosis, and/or the VEGFR-1 signaling network may contribute to the differences observed between the control and saccharin groups. To further explore this hypothesis, we isolated cells separately from the retina and the RPE/choroid segment of the outer retina, extracted mRNA, synthesized cDNA, and performed qPCR analysis for fibrosis-related targets (*Tgfb1*, *Acta2*, and *Smad2*), inflammation-related targets (*Tnf*, *Cd11b*, and *Mcp-1*, and complement factors C3 and C5), and VEGF signaling network-related targets (*Vegfa*, *Vegfb*, *Pgf*, and soluble VGFR1 *sFlt1*). In general, no significant differences were found between the control and saccharin groups for most of these targets in the retina and RPE/choroid samples (refer to Supplementary Fig. S1). However, we did observe differences in certain genes associated with inflammation and the VEGF signaling network (see Fig. 6C). Specifically, there appears to be a trend indicating that AMD-relevant factors of the complement system, *C3* and *C5*, may have reduced expression in the RPE/choroid sample of the saccharin group in comparison to the control (see Supplementary Fig. S1A). Within the VEGF signaling network group, there seems to be a suggestive decrease in the expression levels of *Vegfa*, *Vegfb* (statistically significant, *P* = 0.0159), *Pgf*, and *sFlt1* specifically in the RPE/choroid sample of the saccharin group (see Fig. 6C). Notably, these four genes are specifically involved in the signaling of the VEGFR-1, which can coordinate inflammation with angiogenesis.^21^^,^^22^ However, further investigation with larger sample sizes would be needed to confirm this observation.

**Figure 7.**
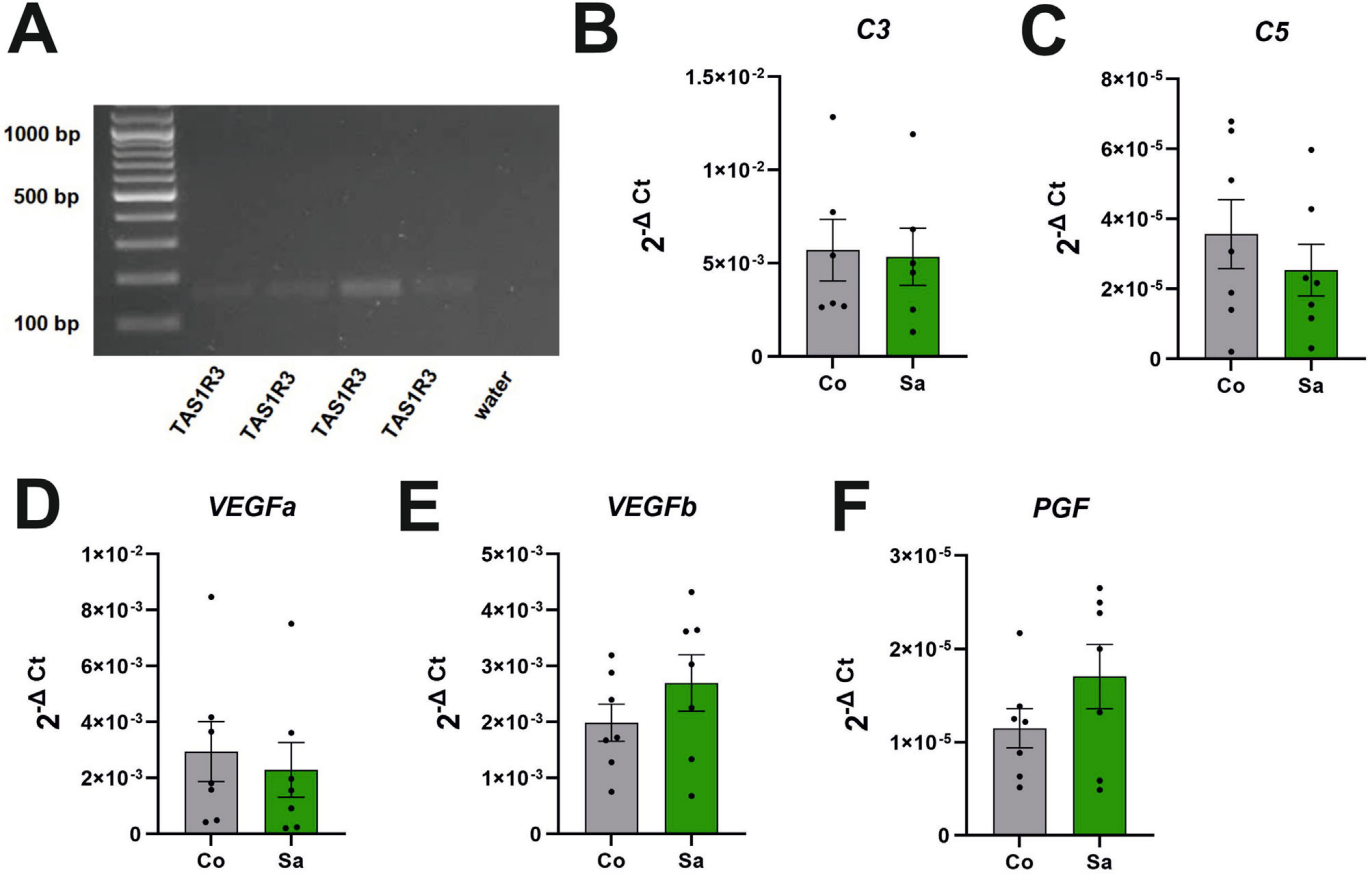
Functional expression of the sweet taste receptor *TAS1R3* in RPE cells. (**A**) Ethidium bromide gel of RT-PCR products from ARPE-19 cells of three different passages showing the *TAS1R3* receptor expression; (**B–F**) qPCR data of gene expression activity after stimulation with 0.03% saccharin in ARPE-19 cells (all data *n* = 7 experiments). Bars represent means with error bars indicating SEM for all graphs in this figure.

In order to provide the saccharin effect at a more mechanistic level, we studied the sweet receptor expression and the reaction of human ARPE-19 cells to saccharin stimulation (Fig. 7). In three passages of the ARPE-19 cell line, we could detect the expression of taste receptor type 1 member 3 (*TAS1R3*) messenger RNA (mRNA) by means of reverse transcription polymerase chain reaction (RT-PCR; see Fig. 7A).

Using qPCR, we found a differential regulation of the genes *VEGF-B* and *PGF* (see Figs. 7B–7F); however, without statistical significance (*VEGF-B*: *P* = 0.2604 and *PGF*: *P* = 0.1944).

## Discussion

To investigate the impact of AS on the course and manifestation of nAMD, we examined 6 common AS metabolites in a cohort of 46 patients with nAMD stratified based on the level of chronic CNV activity under anti-VEGF IVT. Our analysis did not reveal a significant difference in cumulative AS levels of six metabolites among different nAMD disease features. However, saccharin, the oldest known AS, stood out as significantly higher in patients with an effectively controlled CNV phenotype and demonstrated less SHRM formation in human patients. This finding suggests a potential reduction in treatment demand in a dose-dependent manner. Results from a translational murine experiment further supported our findings, as mice fed with saccharin exhibited reduced CNV activity characterized by decreased leakage and bleeding. This effect may be attributed to a reduction in VEGF-mediated immune cell accumulation and inflammation.

Our study reveals that a cumulative use of AS is not significantly beneficial, nor detrimental for the nAMD disease course, but tends to be slightly more protective in the sense of more desirable outcomes in morphology and function for all logged features (although not significant; see Fig. 1E). However, we observe with statistical robustness that individual AS metabolites significantly feature on morphological nAMD conditions in our cohort (see Figs. 2, 3). Although patients with higher levels of saccharin, or another ASs, do not show a significant improvement in visual acuity (refer to Fig. 2E), they do exhibit less SHRM formation and a significantly better outcome in terms of therapy frequency, requiring fewer anti-VEGF IVT (refer to Fig. 3). With current regimens often involving monthly treatments per eye, advances that could reduce the frequency of treatments would not only greatly alleviate the burden on individual patients but also ease the strain on the healthcare system and reduce costs. This potential therapeutic benefit could represent a significant shift in the management of conditions treated with anti-VEGF IVT. Less SHRM formation is generally considered as a positive prognostic biomarker for functional outcome in patients with nAMD under anti-VEGF IVT.^23^ The observation of higher saccharin levels in patients with an effectively controlled CNV activity is dose-depending, that is, patients with higher blood levels yield superior CNV control. This indicates that not a general metabolic conversion in the sense of a reduction of sugar uptake and replacement by AS, but rather the individual molecular profile of some AS effects given features. In fact, on a molecular level, different AS vary fundamentally in their structure and biokinetics, and thus have distinct impact on the human body.^4^^,^^24^ Mechanistically, saccharin stimulates the sweet taste receptor T1R3 for triggering sweet taste.^4^^,^^25^ Besides its well-known expression on the tongue and palate, T1R3 plays a pivotal role in multiple organ systems, including the vasculature where it is a known mitigator of VEGF-induced pathologies: Several studies highlighted the beneficial effect of T1R3 activation by saccharin or sucralose on permeability and leakage, inter alia in the lungs, and the kidneys.^12^^,^^14^^,^^26^ One in vitro study already identified T1R3 activation attenuating VEGF-induced vascular disease of the retina, but in a diabetic context.^13^ Indeed, our data demonstrate the presence of TAS1R3 in the RPE, and it appears that saccharin stimulation of T1R3 may be associated with a decrease in VEGFR-1 signaling (see Fig. 7). With a given nAMD understanding as a VEGF-induced vasculopathy,^2^^,^^3^^,^^27^^–^^29^ this might explain why patients in our cohort with quantitatively higher saccharin blood levels achieve better suppression of their CNV under treatment.

To further investigate this potential mechanistic effect of saccharin on CNV on a molecular level, we conducted an interventional study using a well-established mouse model that closely resembles CNV in AMD: the laser-induced model.^17^^,^^18^ In our study, mice were administered 0.03% saccharin through drinking water at a concentration equivalent to levels found in dietary sources such as lemonade or fruit jam. This concentration results in stable saccharin levels in the blood without affecting urine production.^30^ Consistent with our findings in humans, the group receiving saccharin treatment exhibited a less severe phenotype of CNV at the fluorescence angiography level, characterized by reduced bleedings, less severe bleeding, and decreased fluorescein leakage (see Fig. 4). Additionally, the scar tissue showed reduced fibrosis, and there was a decrease in the migration of mononuclear phagocytes into the neovascular area in the saccharin group. We observed a reduction in the gene expression of complement factors and genes associated with Vegfr-1 signaling (*Pgf*, *Vegfb*, *Vegfa*, and soluble Vegfr1 receptor *sFlt1*) specifically in the RPE/choroid probe, but not in the retinal probe (see Figs. 6, 7, Supplementary Fig. S1).^21^^,^^22^^,^^31^^–^^33^ It is worth noting that we observed these changes in gene expression exclusively in the RPE/choroid sample and, to a lesser extent, in the retinal sample, which displayed minor alterations in response to laser treatment. This observation can be attributed primarily to the laser's specific role in disrupting the outer blood-retina barrier, simulating a scenario akin to the loss of barrier integrity resulting from RPE cell loss in AMD. Moreover, the distinctive response observed in the RPE/choroid sample, as compared to the retinal sample, lends support to our conclusion that saccharin's effects are influenced by saccharin levels in the choroidal bloodstream. This reaction may originate from the RPE, as it has the capability to interact with saccharin in the bloodstream via T1R3 receptors (refer to Fig. 7A). However, it is also plausible that other cell types, such as endothelial cells or immune cells, contribute to the saccharin effects. Based on these observations, we hypothesize that saccharin leads to a reduction in cellular inflammation by attenuating VEGFR-1 signaling and complement activity. This hypothesis aligns with previous studies that have shown a correlation between mononuclear phagocyte accumulation in the outer retina, neovascular area size, and tissue damage severity.^17^^–^^19^^,^^34^^–^^37^ In this scenario, there is a different functional reaction between the receptors VEGFR-1 and VEGFR-2. It seems that VEGFR-2 mainly promotes the VEGF-A-driven endothelial cell proliferation and migration whereas VEGFR-1 seems to combine both endothelial proliferative activity and inflammatory reaction. Thus, both our findings and those of other researchers indicate that inhibiting VEGFR-1 activity, achieved through neutralization of its specific agonist PGF, reduces the neovascular phenotype in the laser-induced model.^19^^,^^34^^,^^35^ It is worth noting that VEGFR-1 is not only expressed in endothelial cells but also in mononuclear phagocytes.^31^^,^^38^ We have demonstrated that the onset of mononuclear phagocyte migration precedes an increase in PGF expression in the laser model, suggesting its involvement as a key trigger.^19^^,^^34^ Another interesting observation is that saccharin intake reduces the expression of *VEGF-B* in the RPE/choroid probe. VEGF-B, along with PGF, acts as a specific agonist of VEGFR-1 and is normally not expressed in healthy mature tissue but becomes expressed after tissue damage.^31^^–^^33^ PGF coordinates blood vessel growth and the innate immune system's response, whereas VEGF-B is involved in balancing proliferation, migration, and maturation of blood vessels and tissue.^21^^,^^33^^,^^39^ The literature on VEGF-B signaling in mononuclear phagocytes is limited and yields divergent results. However, the reduction in *VEGF-B* expression may have a significant impact on endothelial stability and the termination of wound healing processes. The in vivo data from saccharin-treated mice, indicating reduced leakage, less bleeding, and milder bleeding severity, suggest that the decreased VEGF-B signaling may contribute to these beneficial effects.

We cannot offer a mechanistic model how saccharin influences the laser-induced neovascularization of the choroid. The understanding of the physiological role of sweet-tasting receptors T1R2/T1R3 in various tissues might add useful information.^13^^,^^14^^,^^40^ Saccharin and other ASs activate these receptors with higher sensitivity than glucose.^40^ Depending on the cell type, various reactions are possible, such as insulin secretion or even a reduced VEGF-A production by endothelial cells.^14^^,^^40^ Thus, it is likely that saccharin exerts its effects by sweet-taste receptors in the outer retina.

Although our approach is a first in vivo indicator for a potential protective role of saccharin in patients with nAMD, it comes with multiple limitations. The sample size, as well as the number of investigated molecules is limited, and we measured AS blood metabolite levels (not oral uptake or urine levels) at a single timepoint. We cannot exclude metabolic differences in oral uptake, excretion or a diurnal rhythm at this point, because saccharin metabolism in the human body is not fully understood, and might be more complex than we think.^25^ Nevertheless, as far as being scientifically proven, saccharin is excreted slowly over several days in humans and mammals,^41^^,^^42^ which supports the concept of our study. However, due to the early study situation, the lack of a precise understanding of saccharin metabolism in humans, as well as potential detrimental health effects of AS – we do not recommend a preventive intervention in patients with nAMD at this point. Future studies should focus on a replication of the current results in an independent nAMD cohort including multiple daily measurements with exact logging of oral uptake, excretion, as well as health side effects. Although the murine laser-induced model for CNV is a widely and long-established standard model that has proven its translational values in a plethora of studies, considerations of limitations are mandatory.^16^^,^^17^ On the first hand, the mouse retina is a rod-dominated retina and does not form the structure in humans, where the disease takes place: the macula. Second, and for a limitation in translation that is more important, is the fact that we have an acute model based on the rupture of the RPE/Bruch's membrane/choroid complex. Thus, the model does not reproduce the chronic nature of the human disease and due to the comparatively large impact the mouse model bases on a stronger inflammatory reaction than in the development of CNV in humans. However, as macular thickness and not the occurrence of the new blood vessels serves as a surrogate parameter of anti-VEGF-A treatments in the human condition, it is likely that also in the treatment of patients with AMD the decision bases on a parameter that is stronger linked to inflammation than on angiogenesis. Many studies support this conclusion by demonstrating a reduction of CNV by inflammation inhibition.^29^^–^^33^

In conclusion, we deem our findings another reference for saccharin (and potentially other AS metabolites) as potential mitigators for neovascular diseases of the eyes, and first clinical evidence for this in nAMD. Our murine model enriches our mechanistic understanding on a molecular level and connects VEGFR-1 signaling with immune signals. Our observations, as well as the general study situation, underlines the need for more – potentially – interventional studies to evaluate the preventive potential of saccharin in nAMD.

## Supplementary Material

Supplement 1

Supplement 2

Supplement 3

Supplement 4
