# Supplementary material for: Exploring the Impact of Saccharin on Neovascular Age-Related Macular Degeneration: A Comprehensive Study in Patients and Mice
Source: Invest Ophthalmol Vis Sci. 2024 Apr 1;65(4):5. doi: 10.1167/iovs.65.4.5 (PMC10996979; doi:10.1167/iovs.65.4.5)
Supplement: Supplement 2 [file iovs-65-4-5_s002.pdf]

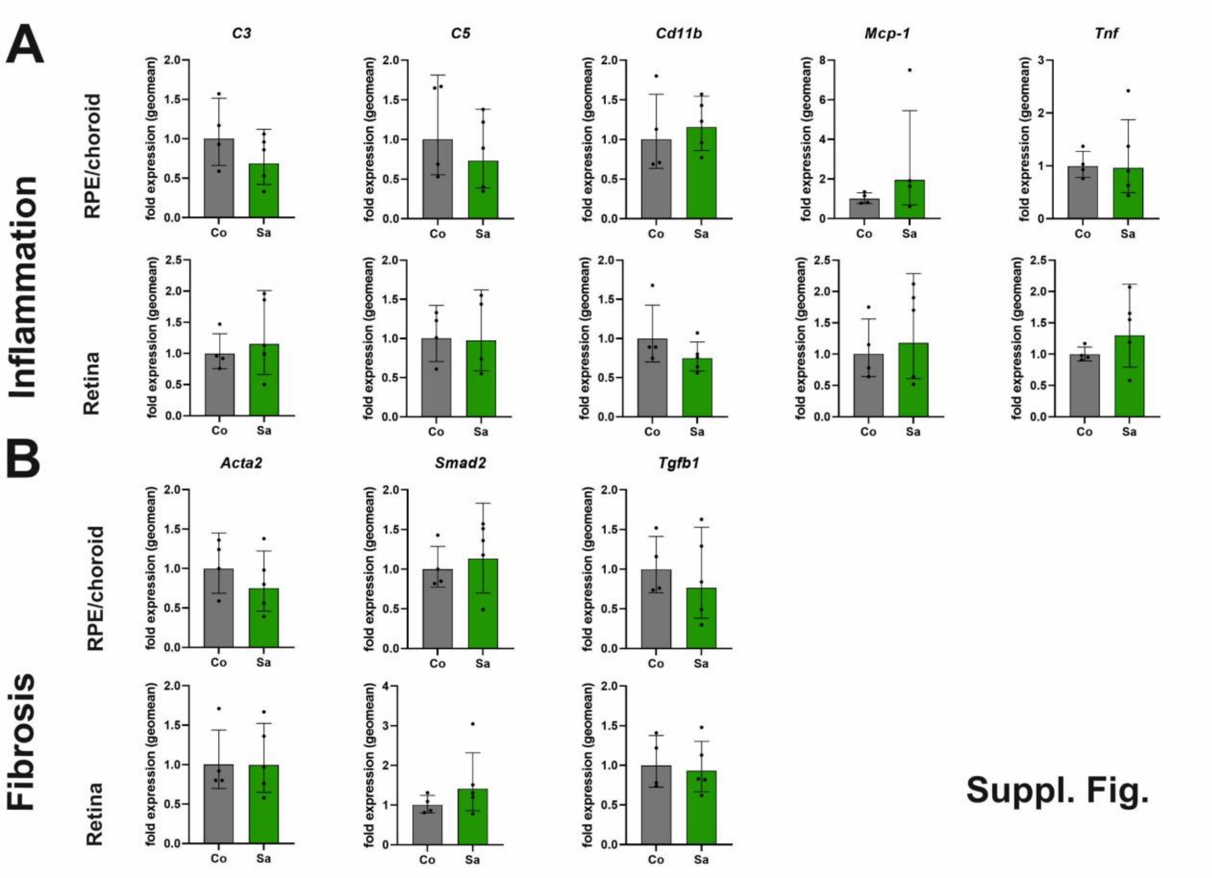

Suppl. Fig. 1.

qPCR to determine differences in the gene expression; mRNA was harvested from either RPE/choroid tissue preparations (upper rows) or retina preparations (lower rows) of mice from the control group (n=4 retinal or RPE/choroid tissue samples, grey bars) and the saccharin group (n=5 retinal or RPE/choroid tissue samples; green bars); the figure shows the data for genes with biological relevance in inflammation: **A** complement factors *C3* and *C5*, *Cd11b*, *Mcp1*, *Tnf* (note both *C3* and *C5* with reduced expression); **B** fibrosis: *αSma*, *Smad2*, *Tgfb1*. Bars represent geomeans with error bars indicating SD for all graphs in this figure.
