# Supplementary material for: Exploring the Impact of Saccharin on Neovascular Age-Related Macular Degeneration: A Comprehensive Study in Patients and Mice
Source: Invest Ophthalmol Vis Sci. 2024 Apr 1;65(4):5. doi: 10.1167/iovs.65.4.5 (PMC10996979; doi:10.1167/iovs.65.4.5)
Supplement: Supplement 3 [file iovs-65-4-5_s003.pdf]

	**Overall cohort**	**Chronically active CNV (CAC)***	**Effectively controlled CNV (ECC)****
N	46	25	21
Age in years (median [IQR])	79.5 [76.0, 83.75]	79.0 [75.0, 82.0]	79.71 [77.0, 85.0]
Sex	23 female, 23 male	10 female, 15 male	13 female, 8 male
Best-corrected visual acuity in LogMAR (median [IQR])	0.46 [0.21, 0.60]	0.48 [0.28, 0.64]	0.46 [0.16, 0.54]

Suppl. Table 1. Demographic features between high and low anti-VEGF intravitreal treatment cohort.
