# Supplementary material for: Exploring the Impact of Saccharin on Neovascular Age-Related Macular Degeneration: A Comprehensive Study in Patients and Mice
Source: Invest Ophthalmol Vis Sci. 2024 Apr 1;65(4):5. doi: 10.1167/iovs.65.4.5 (PMC10996979; doi:10.1167/iovs.65.4.5)
Supplement: Supplement 4 [file iovs-65-4-5_s004.pdf]

Primary and secondary antibodies				
**Antigen**	**host**	**dilution**	**Cat#**	**Company**
F-actin (Acti-stain 555 phalloidin)	mushroom Amanita phalloides	1:300	PHDH1	Cytoskeleton, Inc.
Vimentin	mouse monoclonal	1:100	sc-32322	Santa Cruz
Iba1	goat polyclonal	1:200	ab5076	Abcam
**Conjugate**	**host and target species**	**dilution**	**Cat#**	**Company**
AF488	rabbit anti mouse	1:400	A11059	Invitrogen
AF405	rabbit anti goat	1:400	ab175667	Abcam

Suppl. Table 2. Methods. Primary and secondary antibodies used for immunohistochemistry.
